# High return to sport after rotator cuff repair in racket sport players

**DOI:** 10.1002/jeo2.70396

**Published:** 2025-09-22

**Authors:** Ayham Jaber, Yazan Jaber, Christopher J. Hawryluk, Silvia Cardarelli, Gianmarco Marcello, Peter J. Millett, Giovanni Di Giacomo, Filippo Familiari

**Affiliations:** ^1^ Department of Orthopaedics Heidelberg University Hospital Heidelberg Germany; ^2^ Steadman Philippon Research Institute Vail USA; ^3^ College of Medicine Almaarefa University Riyadh Saudi Arabia; ^4^ Department of Orthopedics and Traumatology Tor Vergata University Rome Italy; ^5^ Department of Orthopedics and Traumatology Fondazione Policlinico Universitario Campus Bio‐Medico Rome Italy; ^6^ The Steadman Clinic Vail Colorado USA; ^7^ Department of Orthopedics and Traumatology Concordia Hospital Rome Italy; ^8^ Department of Orthopedics Magna University of Catanzaro Catanzaro Italy

**Keywords:** racket sports, return to sport, rotator cuff, shoulder, tennis

## Abstract

**Purpose:**

To assess the return‐to‐sport (RTS) rates and outcomes in racket sports players after isolated supraspinatus full thickness tears, and to investigate the influence of patient‐, injury‐, and treatment‐specific factors on these outcomes.

**Methods:**

This retrospective study reviewed 38 racket sport players (tennis and padel) who underwent primary arthroscopic RCR performed by the senior author (G.D.G.). Inclusion criteria included patients aged 16 years or older with a history of rotator cuff tear, playing racket sports at least weekly prior to injury, and a minimum two‐year follow‐up. Functional outcomes were assessed using the Visual Analogue Scale for pain (VAS‐score) and the 12‐Item Short Form Survey (SF‐12). RTS was defined as returning to the same or higher level. Statistical analysis was performed to analyse categorical and continuous variables.

**Results:**

Ninety percent all participants were male, with a mean age of 58 years. The majority (95%) played racket sports recreationally, with tennis being the most common sport. Most participants (80%) played on clay courts. Thirty‐four participants (85%) returned to sport, with 69% returning at the same level and 10% at a higher level. The median postoperative VAS score was 0 (IQR 0–0). Patient satisfaction was high, with 97.5% of patients reporting satisfaction. No significant differences in RTS were found based on injury mechanism or symptom duration, though patients with traumatic injuries reported higher mental component summary scores.

**Conclusion:**

Most racket sport players who undergo arthroscopic RCR for full‐thickness supraspinatus tears are able to return to sport, with most returning at the same or higher level of play. Trauma‐related injuries may positively influence mental recovery compared to degenerative injuries. Further prospective studies with larger cohorts and a greater focus on competitive athletes are needed to better predict patient outcomes.

**Level of Evidence:**

Level IV.

**Abbreviations**
IQR: interquartile range
MCS: mental component summary
PCS: physical component summary
PROMs: Patient‐Reported Outcome Measures
RC: rotator cuff
RCR: rotator cuff repair
ROM: range of motion
RTS: return to sport
SF‐12: 12‐Item Short Form Survey
VAS: Visual Analogue Scale for pain
WOSI: Western Ontario Shoulder Instability Index

## INTRODUCTION

Returning to racket sports after rotator cuff repair (RCR) is a major concern for both competitive athletes and recreational players, as rotator cuff (RC) injuries are a common shoulder pathology that often cause pain and dysfunction, frequently requiring surgical interventions. The supraspinatus tendon is most commonly involved [11]. In the general population, the prevalence of rotator cuff tears is estimated to range from 9.7% in individuals aged 20 years to 62% in those aged 80 years [14]. The prevalence is relatively high among athletes. A study by Kaplan and colleagues found that 12% of competitive American football players had a history of rotator cuff injuries [7]. Return‐to‐sport (RTS) rates in recreational athletes are generally lower than in competitive athletes. In particular, overhead athletes show a lower RTS rate than in other sports [3]. The time frame for returning to racket sports can vary, but many athletes are able to return to their sport within 6 to 14 months post‐surgery, depending on the severity of the tear and the success of the treatment [2, 3, 8].

Factors influencing RTS include the size of the rotator cuff tear, the type of repair performed, and the player's adherence to the rehabilitation protocol. High‐level overhead athletes, such as tennis players, may face a lower rate of return to their previous level of play compared to recreational athletes, with only about 50% returning to their pre‐injury level [3, 8, 12]. Racket sports, such as tennis and padel, also involve high‐frequency, high‐velocity overhead motions and demand precise control and power during strokes, placing significant stress on the rotator cuff muscle group. Specific biomechanical and physiological demands of racket sports have been previously reported, including the impact on shoulder range of motion (ROM), strength and serve speed [6, 9]. We aim to report the outcomes and RTS rates of racket sport players (tennis, padel) following RCR and identify patient‐, injury‐ and treatment‐specific factors that may influence this outcome. We hypothesise that repair of full‐thickness supraspinatus tears leads to a high RTS in racket sport players with a low rate of revision surgery.

## METHODS

### Patient selection

This was a retrospective review of racket sports players who underwent primary arthroscopic repair of isolated full‐thickness supraspinatus tears, using a single‐row technique, performed by the senior author (G.D.G.). Patients were retrospectively followed up to collect Patient‐Reported Outcome Measures (PROMs) to assess RTS and postoperative performance.

Inclusion criteria were (1) ≥ 16 years old at the time of surgery, (2) primary rotator cuff repair for an isolated full‐thickness supraspinatus tear (3) played racket sports at least once a week in training or in a match prior to the injury, (4) minimum 2‐year follow‐up and (5) ability to communicate with healthcare professionals and give valid informed consent.

Exclusion criteria included revision surgery, preexisting connective tissue disorders, associated fractures, and lack of consent for follow‐up.

Demographic data were collected, including age, sex, race, body mass index, smoking status, prior conservative treatments, type of racket sport, playing surface (grass, clay, hard, or artificial grass), level of play (competitive or recreational), mechanism of injury (purely traumatic or purely degenerative), symptoms duration in months, number of anchors, duration of brace use (in weeks) and rehabilitation protocol. We defined ‘trauma‐related’ injury as any acute injury event confirmed by patient history (vs. chronic/insidious onset). We defined ‘degenerative’ as tears without a clear traumatic onset.

Conservative treatment included physical therapy and/or corticosteroid joint injections. Duration of therapy and the use of corticosteroid was based on the severity of symptoms and discretion of the surgeon and patient.

The study was approved by the institutional review board (Calabria Regional Ethics Committee—111/2025) and was conducted in accordance with the Helsinki Declaration of 1975, as revised in 2013. All patients provided informed consent for participation in the study, and follow‐up was collected through surveys.

### Surgical technique

The patient is positioned in the beach‐chair position, and the operative arm is secured using a pneumatic arm holder. Standard arthroscopic portals—posterior, lateral, and anterior‐superior, are established. A diagnostic arthroscopy is performed to evaluate intra‐articular pathology, followed by thorough assessment of the rotator cuff tear.

After transitioning to the subacromial space, a meticulous bursectomy is performed to enhance visualization of the rotator cuff tendons. The torn tendon is then prepared by debriding frayed, scarred, or devitalized tissue using motorized shavers and curettes. Simultaneously, the bony footprint is prepared to promote tendon healing, and microfracture may be utilized if bone quality is compromised.

Attention is then directed to anchor placement. A single‐row repair is performed, with one to two suture anchors used depending on tear size and morphology. Pilot holes are created using specialised drill guides, ensuring appropriate depth and trajectory. Anchors, either titanium alloy or bioabsorbable, are inserted into the pilot holes with secure fixation in the cancellous bone. Each anchor is loaded with two or three high‐strength sutures.

Sutures are passed through the tendon using arthroscopic suture passers, with the configuration, simple stitches or mattress variants, determined by the tendon involved and tear pattern. For supraspinatus repairs, simple or horizontal mattress sutures are used. Once all sutures are passed, they are tied arthroscopically using knot pushers and knot tiers. Each knot is secured with a sequence of seven half‐hitches to minimise the risk of slippage, with tension carefully adjusted to optimise tendon‐to‐bone contact without overtightening. The repair is evaluated to ensure it adequately restores the anatomical footprint and avoids excessive tension or gapping.

Upon completion, the repair is assessed under direct arthroscopic visualization with gentle passive range of motion to evaluate stability and rule out impingement. The tendon should remain securely fixed without excessive movement. All instruments are then withdrawn, portals are closed using appropriate skin closure techniques, and a sterile dressing is applied.

### Rehabilitation protocol

Postoperative physiotherapy after arthroscopic rotator cuff repair is adjusted based on the management of the long head of the biceps tendon. For patients who underwent biceps tenotomy, the first 25 days focus on immobilisation, assisted elbow extension, and scapulothoracic muscle isometric exercises. From days 25 to 60, supervised therapy includes gentle passive and assisted shoulder movements in the scapular plane, scapular strengthening, and light rotator cuff exercises performed below 90° of abduction, while avoiding elastic resistance. After day 60, closed kinetic chain exercises above 90°, proprioceptive training, and eccentric cuff strengthening are added, along with postural training.

For patients who underwent subpectoral biceps tenodesis, early rehab focuses on passive shoulder mobility (scapular and frontal planes) and allows passive elbow extension from day 7. Days 25–60 follow a similar protocol to tenotomy but allow earlier motion above 90° (without rotation) and more emphasis on closed chain scapular and cuff co‐contraction. From day 60, short‐lever elastic strengthening and proprioceptive/plyometric exercises are used, avoiding long‐lever open chain movements, and ending with postural training.

### Outcome assessment

The primary outcome assessment was RTS. Patients reported whether they returned to racket sports, and whether they played at the same, higher, or lower level than before the injury. Outcomes were also assessed using the Visual Analogue Scale for pain (VAS‐Pain), which ranges from 0 to 10, with higher scores indicating greater pain, [1] and the 12‐Item Short Form Survey (SF‐12) [13], a 12‐item questionnaire used to assess generic health outcomes from the patient's perspective. Two summary scales (the physical and the mental component summary, PCS and MCS, respectively) [15, 16] can be calculated with scores ranging from 0% to 100%, with higher scores indicating a better quality of life. The assessments were performed at follow‐up using standardised surveys.

Patient satisfaction was reported using a 5‐point Likert scale [5], as follows: ‘very satisfied’, ‘satisfied’, ‘neither satisfied nor dissatisfied’, ‘dissatisfied’ and ‘very dissatisfied’. Reoperations and complication were also reported.

### Statistical analysis

Data were analysed using SPSS (IBM Corp., Ver. 27.0, New York, USA), with categorical variables being presented as frequencies with percentages, and continuous variables presented as mean values (with standard deviation) or median values (with interquartile range)—based on the pattern of distribution. The Shapiro–Wilk test was used to assess the normality of data, while histograms were used for visual confirmation. Comparison between categorical variables was performed using the chi‐square test. For non‐normal continuous variables, the Mann–Whitney *U* test was used when there were two comparator groups, and the Kruskal–Wallis test was used for more than two comparator groups (number of anchors). For normally distributed variables, the independent samples t‐test was planned. A multivariate analysis model was constructed to investigate predictive factors for RTS time, VAS, PCS, and MCS scores, with age, sex, and BMI being the covariates. All analyses were conducted at a 5% level of significance.

## RESULTS

**Figure 1:**
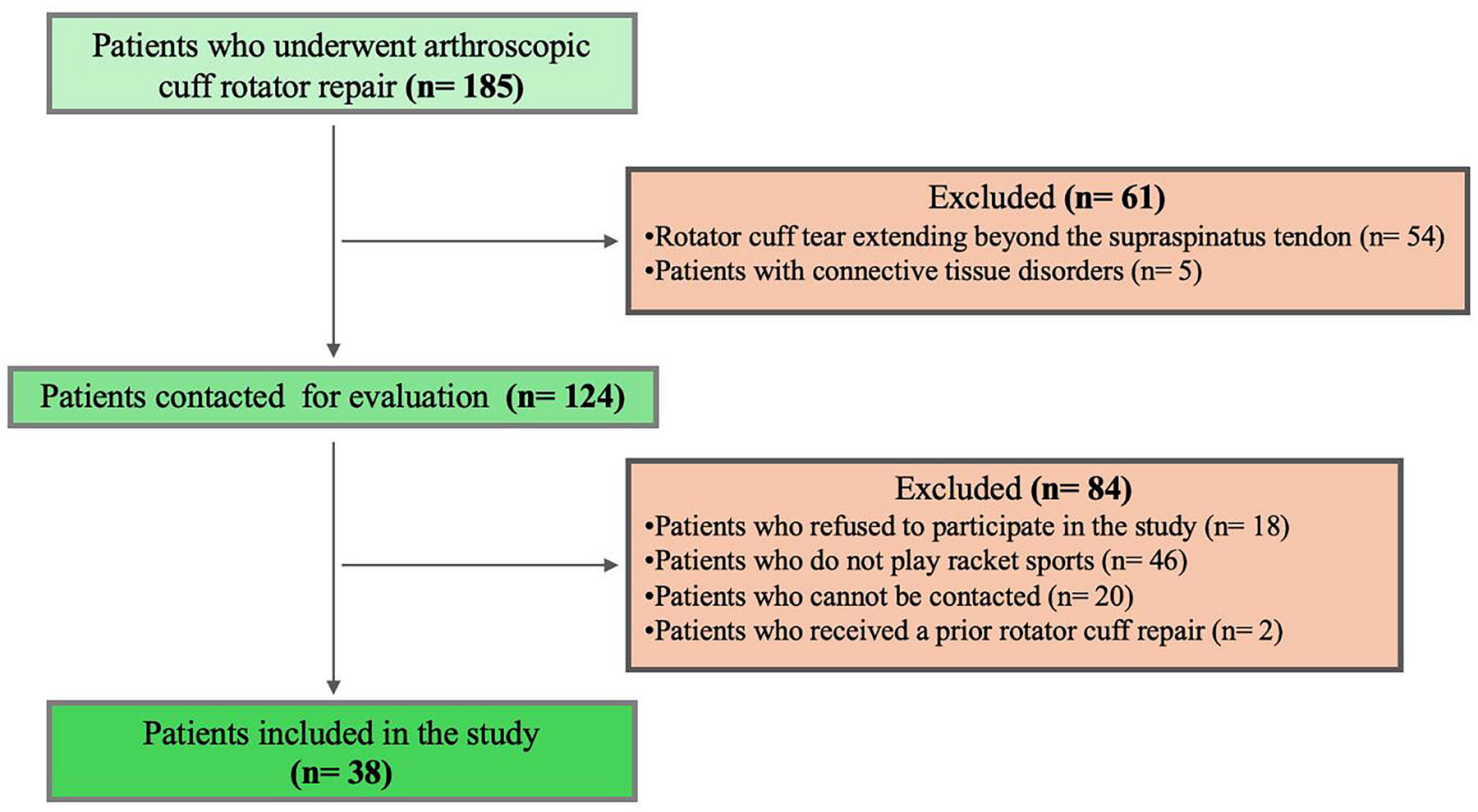
Flowchart of all patients included after applying the exclusion and inclusion criteria.

The study included a total of 38 participants in the final cohort (Figure 1), the majority of whom were males (approximately 90%). The mean age of the participants was just under 58 years and in terms of laterality, 76% of them were right‐handed. Four participants were current smokers while seven were former smokers. The median follow‐up in months was 32.5 (27.5–42.5). A detailed breakdown of participant characteristics is outlined in Table 1.

**Table 1:** Patient demographics.

Characteristic	Value
Mean age ± SD	57.7 ± 10.1
Gender (*n*, %)	
‐ Male	34 (89.5)
‐ Female	4 (10.5)
Laterality (*n*, %)	
‐ Right	29 (76.3)
‐ Left	9 (23.7)
Dominant arm (*n*, %)	
‐ No	10 (26.3)
‐ Yes	28 (73.7)
Mean height in m ± SD	1.7 ± 0.1
Mean weight in kg ± SD	78.5 ± 12.2
Mean BMI (kg/m^2^) ± SD	25.7 ± 2.8
Previous conservative treatment (*n*, %)	19 (50.0)
Smoking status (*n*, %)	
‐ Never smoked	27 (71.1)
‐ Current smoker	4 (10.5)
‐ Former smoker	7 (18.4)
Race (*n*, %)	
‐ White	37 (97.4)
‐ Black	1 (2.6)
Concomitant procedures (*n*, %)	
‐ Acromioplasty	4 (10.5)
‐ Bursectomy	32 (84.2)
‐ Arthrolysis	21 (55.3)
‐ Biceps Tenotomy	20 (52.6)
‐ Biceps Tenodesis	32 (84.2)
‐ Mumford procedure	1 (2.6)
Median follow up time in months (IQR)	32.5 (27.5–42.5)

Abbreviations: BMI, body mass index; IQR, interquartile range; SD, standard deviation.

**Figure 2:**
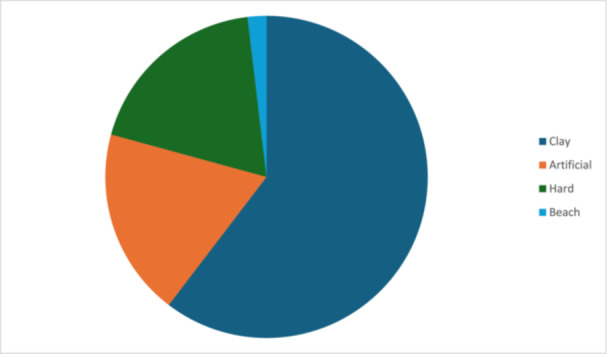
Pie chart showing distribution of participants based on type of surface.

Analysis of sport characteristics showed that 28 participants played only tennis, four played only padel, and five played both. 95% played their sport at a recreational level and in terms of type of surface, clay was the most common surface, used by 79% of the participants (Figure 2). Purely traumatic injuries were seen in 15 participants, while 23 had purely degenerative injuries. The median symptom duration was 4.0 months (Table 2). In terms of number of anchors, 1 was the most common, being used in more than half of the cohort, while three anchors were used in only one participant (Figure 2). A total of 33 participants reported RTS, with approximately 74% returning at the same level, while three participants (9%) returned at a higher level. Complications following the procedure were observed in three patients, and all except one participant reported satisfaction (Table 3).

**Table 2:** Sport and injury characteristics.

Characteristic	Value
Type of sport (*n*, %)	
‐ Tennis	28 (73.7)
‐ Padel	4 (10.5)
‐ Both tennis and padel	5 (13.2)
‐ Beach tennis	1 (2.6)
Level of play (*n*, %)	
‐ Competitive	2 (5.2)
‐ Recreational	36 (94.7)
Type of surface (*n*, %)	
‐ Clay	30 (78.9)
‐ Artificial grass	8 (21.1)
‐ Hard	9 (23.7)
‐ Sand	1 (2.6)
Mechanism of injury (*n*, %)	
‐ Traumatic	15 (39.5)
‐ Degenerative	23 (60.5)
Median symptom duration in months (IQR)	4.0 (1.0–12.0)

Abbreviation: IQR, interquartile range.

**Table 3:** Patient outcomes.

Characteristics	Value
RTS (*n*, %)	33 (86.8)
RTS level (*n*, %)	
‐ Same	25 (73.5)
‐ Higher	3 (8.8)
‐ Lower	6 (17.6)
Median RTS time in months (IQR)	8.0 (6.0–12.0)
Median VAS score (IQR)	0.0 (0.0–2.0)
Median PCS (IQR)	53.6 (51.5–55.2)
Median MCS (IQR)	58.4 (56.2–60.7)
Complication (*n*, %)	3 (7.9)
Patient satisfaction (*n*, %)	
‐ Yes	37 (97.3)
‐ No	1 (2.7)

Abbreviations: IQR, interquartile range; MCS, mental component summary; PCS, physical component summary; RTS, return‐to‐sport; VAS, Visual Analog Scale for pain.

Table 4 shows the results of the analysis examining associations between study parameters and the mechanism of injury. The analysis showed no statistically significant differences between mechanism of injury based on RTS level, median RTS time, VAS score, and median PCS score. For MCS, however, those with purely traumatic injuries had higher median scores than those with purely degenerative injuries (60.7 vs. 57.8), with statistical significance (*p* = 0.017). Meanwhile, no statistically significant associations were found between the tested study parameters and duration of symptoms (grouped into less than or 4 months vs. more than 4 months) (Table 5), and for the comparison of study parameters based on number of anchors used (Table 6).

**Table 4:** Associations between study parameters and mechanism of injury.

Parameters	Purely traumatic	Purely degenerative	*p*‐value
RTS level			0.637
‐ Same	11 (73.3)	14 (60.9)	
‐ Higher	2 (13.3)	1 (4.3)	
‐ Lower	2 (13.3)	4 (17.4)	
Median RTS time in months (IQR)	6.0 (6.0–12.0)	8.0 (6.0–12.0)	0.465
VAS score	0.0 (0.0–0.0)	0.0 (0.0–0.0)	0.675
Median PCS (IQR)	53.5 (50.0–55.2)	53.8 (51.6–55.0)	0.742
Median MCS (IQR)	60.7 (58.7–60.8)	57.8 (53.3–59.7)	0.012*

Abbreviations: IQR, interquartile range; MCS, mental component summary; PCS, physical component summary; RTS, return‐to‐sport; VAS, Visual Analog Scale for pain.

**Table 5:** Comparison of study parameters based on duration of symptoms.

Parameters	Less than or 4 months	More than 4 months	*p*‐value
RTS level			0.069
‐ Same	17 (85.0)	8 (57.1)	
‐ Higher	2 (10.0)	1 (7.1)	
‐ Lower	1 (5.0)	5 (35.7)	
Median RTS time in months (IQR)	6.0 (6.0–16.5)	8.0 (6.8–10.5)	0.314
VAS score	0.0 (0.0–0.0)	0.0 (0.0–0.0)	0.185
Median PCS (IQR)	53.6 (50.0–55.2)	53.5 (52.9–55.0)	0.724
Median MCS (IQR)	59.3 (57.3–60.7)	57.6 (54.1–60.3)	0.257

Abbreviations: IQR, interquartile range; MCS, mental component summary; PCS, physical component summary; RTS, return‐to‐sport; VAS, Visual Analog Scale for pain.

**Table 6:** Comparison of study parameters based on number of anchors used.

Parameters	1	2	3	*p*‐value
RTS level				0.577
‐ Same	13 (65.0)	11 (84.6)	1 (100)	
‐ Higher	3 (15.0)	0	0	
‐ Lower	4 (20.0)	2 (15.4)	0	
Median RTS time in months (IQR)	8.0 (6.0–12.0)	8.0 (6.0–8.0)	4.0 (4.0–4.0)	0.204
VAS score	0.0 (0.0–0.0)	0.0 (0.0–0.0)	0.0 (0.0–0.0)	0.891
Median PCS (IQR)	53.8 (53.0–55.0)	52.9 (50.2–55.3)	54.9 (54.9–54.9)	0.630
Median MCS (IQR)	58.0 (55.8–60.8)	59.0 (53.8–60.7)	56.7 (56.7–56.7)	0.753

Abbreviations: IQR, interquartile range; MCS, mental component summary; PCS, physical component summary; RTS, return‐to‐sport; VAS, Visual Analog Scale for pain.

Results of the multivariate analysis after controlling for age, sex, and BMI revealed no statistically significant associations between the tested independent variables (mechanism of injury, number of anchors, and symptom duration), and dependent variables (RTS time, VAS score and MCS score) as seen in Table 7.

**Table 7:** Multivariate analysis for correlation with RTS time, VAS score, PCS score and MCS score (controlling for age, sex and BMI).

Variables	Standardised coefficient (*B*)	95% CI	*p*‐value
RTS time			
Purely traumatic injury	−0.03	−6.23 to 5.43	0.890
Number of anchors	−1.16	−7.68 to 5.43	0.467
Symptom duration	−1.17	−0.29 to 0.12	0.419
VAS score			
Purely traumatic injury	−0.18	−1.07 to 0.34	0.348
Number of anchors	0.01	−0.69 to 0.72	0.968
Symptom duration	0.01	−0.03 to 0.03	0.989
PCS score			
Purely traumatic injury	0.14	−1.48 to 3.64	0.396
Number of anchors	0.15	−1.43 to 3.50	0.399
Symptom duration	−0.20	−0.15 to 0.04	0.248
MCS score			
Purely traumatic injury	−0.27	−5.49 to 0.47	0.096
Number of anchors	−0.15	−4.12 to 1.62	0.383
Symptom duration	−0.05	−0.09 to 0.13	0.742

Abbreviations: CI, confidence interval; IQR, interquartile range; MCS, mental component summary; PCS, physical component summary; RTS, return‐to‐sport; SF‐12: 12‐Item Short Form Survey; VAS, Visual Analog Scale for pain.

## DISCUSSION

The main finding in our study was that approximately 80% of racket sport players who underwent arthroscopic RCR for full‐thickness supraspinatus tears successfully returned to their sport, with the majority returning to the same or higher level of performance. Furthermore, most patients reported being pain‐free and satisfied with their surgical outcome. Neither the duration of symptoms nor the number of anchors used had a statistically significant impact on RTS success or validated functional outcome scores. Interestingly, tear etiology appeared to influence mental health outcomes, with trauma‐related injuries associated with better mental component scores (MCS) on the SF‐12 survey. These findings suggest that patients with trauma‐related injuries experience more favorable mental health outcomes postoperatively. This aligns with the literature indicating that a well‐defined injury event can enhance psychological engagement in rehabilitation, whereas chronic, degenerative conditions may contribute to prolonged stress and decreased motivation during recovery [4].

The improvement in functional scores postoperatively aligns with prior literature on rotator cuff repair outcomes in athletes. In a systematic review, Altintas et al. reported that 73.3% of recreational athletes and 61.5% of competitive athletes were able to return to sport after arthroscopic rotator cuff repair, but only 38% of overhead athletes returned to their pre‐injury level of sport after RCR in partial to full‐thickness tears [3]. The reason for the lower return to play is not entirely clear. The analysis, however, included not only racket sport athletes but players of other overhead sports. The analysis looked at both recreational and competitive athletes. In our cohort, only one patient played competitively, so the cohort largely composed of recreational athletes. This disparity highlights the impact of surgical recovery and rehabilitation on athletic performance, particularly in sports that place high demands on shoulder endurance. Klouche and colleagues reported an overall RTS rate of 84.7% across various sports, including tennis, with 65.9% returning to an equivalent level of play [8]. Specifically, for tennis players, the RTS rate was included in the overall analysis but not separately detailed. In younger athletes under 45 years, Moussa et al. reported a cumulative RTS rate of 75.2%, with 56.1% returning to the same or higher level, indicating better outcomes in younger populations [10]. However, the specific breakdown for racket sports versus other sports was not provided.

The present study focused on a specific population, racket sport players, which is underrepresented in the current literature. All surgeries were performed by a single surgeon using one technique to address a full‐thickness supraspinatus tear. Due to the retrospective design of the study, preoperative data and outcomes concerning improvement could not be reported. Our sample size was relatively small, which may limit generalisability. The exact tear characteristics and other imaging findings were not included. We selected patients with isolated supraspinatus tears to create a homogeneous study group. Yet excluding larger/multi‐tendon tears may further limit generalisability. A reliable statement about RTS in competitive athletes cannot be made since most of our cohort included recreational athletes. Furthermore, because this was a retrospective study, we did not formally measure patient adherence to the rehabilitation protocol.

## CONCLUSION

Most racket sport players who undergo arthroscopic RCR for full‐thickness supraspinatus tears are able to return to their sport, with the majority returning to the same or a higher level of play. The duration of symptoms and the number of anchors used do not seem to significantly affect outcomes. However, the findings suggest that trauma‐related injuries may have a more positive influence on mental recovery compared to degenerative injuries. Further prospective studies with larger cohorts and a greater focus on competitive athletes are needed to better predict these outcomes.

## AUTHOR CONTRIBUTIONS

All authors whose names appear on the submission made substantial contributions to the conception or design of the work; or the acquisition, analysis, or interpretation of data; or the creation of new software used in the work; drafted the work or revised it critically for important intellectual content; approved the version to be published; and agree to be accountable for all aspects of the work in ensuring that questions related to the accuracy or integrity of any part of the work are appropriately investigated and resolved.

## CONFLICT OF INTEREST STATEMENT

Ayham Jaber, Christopher J. Hawryluk, and Peter J. Millett: The above authors' positions are supported by the Steadman Philippon Research Institute, which is a 501(c)(3) non‐profit institution supported financially by private donations and corporate support. The Steadman Philippon Research Institute (SPRI) exercises special care to identify any financial interests or relationships related to research conducted here. During the past calendar year, SPRI has received grant funding or in‐kind donations from Arthrex, Canon, DJO, Icarus Medical, Medtronic, Ossur, Smith&Nephew, SubioMed, and Stryker & Wright Medical. Filippo Familiari, Yazan Jaber, Silvia Cardarelli, Gianmarco Marcello, and Giovanni Di Giacomo: The above authors, their immediate family, and any research foundation with which they are affiliated did not receive any financial payments or other benefits from any commercial entity related to the subject of this article. Peter J. Millett: The above author receives royalties, consultant payments, and research support from Arthrex which is not related to the subject of this work. Dr. Millett also owns stock options with VuMedi.

## ETHICS STATEMENT

The study was approved by the institutional review board (Calabria Regional Ethics Committee – 111/2025) and was conducted in accordance with the Helsinki Declaration of 1975, as revised in 2013. All patients provided informed consent for participation in the study, and follow‐up was collected through surveys. All patients provided informed consent for participation in the study.

## Data Availability

Data is available upon reasonable request.
